# Human Brain Mapping of Visual Script Familiarity between Phonological and Logographic Language: 3 T Functional MRI Study

**DOI:** 10.1155/2017/5732642

**Published:** 2017-07-05

**Authors:** Nambeom Kim, Jongho Kim, Chang-Ki Kang, Chan-A Park, Mi-Ra Lim, Young-Bo Kim, Byung-Gee Bak

**Affiliations:** ^1^Neuroscience Research Institute, Gachon University, Incheon, Republic of Korea; ^2^Department of Radiology, Johns Hopkins University School of Medicine, Baltimore, MD, USA; ^3^Department of Radiological Science, College of Health Science, Gachon University, Incheon, Republic of Korea; ^4^Bioimaging Research Team, Division of Bioconvergence Analysis, Korea Basic Science Institute, Ochang, Cheongju, Republic of Korea; ^5^Department of General Education, Ajou University, Seoul, Republic of Korea; ^6^Department of Neurosurgery, Gachon University Gil Hospital, Incheon, Republic of Korea; ^7^Department of Education, Chonbuk National University, Jeonju, Republic of Korea

## Abstract

Neurolinguistic circuitry for two different scripts of language, such as phonological scripts (PhonoS) versus logographic scripts (LogoS) (e.g., English versus Chinese, resp.), recruits segregated neural pathways according to orthographic regularity (OrthoR). The purpose of this study was to identify the effect of VSF for cortical representation according to different OrthoR to represent Hangul versus Hanja as PhonoS versus LogoS, respectively. A total of 24 right-handed, native Korean undergraduate students with the first language of PhonoS and the second language of LogoS were divided into high- or low-competent groups for L2 of LogoS. The implicit word reading task was performed using Hanja and Hangul scripts during functional magnetic resonance imaging (fMRI) acquisition. Fluctuations of fMRI BOLD signal demonstrated that the LogoS was associated with the ventral pathway, whereas PhonoS was associated with the dorsal pathway. By interaction analysis, compared with high-competent group, low-competent group showed significantly greater activation for Hanja than for Hangul reading in the right superior parietal lobule area and the left supplementary motor area, which might be due to neural efficiency such as attention and cognition rather than core neurolinguistic neural demand like OrthoR processing.

## 1. Introduction

Neurolinguistic circuitry for two different scripts of language, namely, phonological script (PhonoS) versus logographic script (LogoS) (e.g., English versus Chinese, resp.), recruits segregated neural pathways according to orthographic regularity (OrthoR) on human brain cortical mapping using functional magnetic resonance imaging (fMRI) [1, 2]. For example, reading PhonoS such as English, Japanese Kana, and Korean Hangul converts grapheme into phoneme as a type of assembled phonology, whereas reading LogoS such as Chinese, Japanese Kanji, and Korean Hanja only retrieves phonology from an internal lexicon as a type of addressed phonology [3, 4]. Neuroimaging studies revealed that PhonoS recruits the left dorsal pathway via the angular gyrus (AG) or supramarginal gyrus (SMG) and involves the ventrolateral prefrontal cortex (VLPFC) and the lower part of Broca's area. On the other hand, LogoS recruits the left ventral pathway via the inferior medial temporal lobe involving the fusiform gyrus (FG) and the pars triangularis/pars opercularis [5–8].

Given the better understanding of neurolinguistic circuitry of PhonoS versus LogoS as depicted with fMRI study, further work investigated whether learning the second language (L2) is related to assimilation versus accommodation with respect to native first language circuitry (L1) [9–12]. Brain areas for L2 stimuli that overlap with those for L1 may be secondary to assimilating or accommodating the new writing system [13]. In this regard, it is very important to stabilize substantial cortical mapping across each script and eliminate many other confounding variables to recruit neural demands. Given neural demands and efficiency as the main determinant on cortical mapping, visual script familiarity (VSF) needs to be considered since it affects not only cortical representation [14–16] but also performance of verbal tasks, for example, picture naming [17], lexical decision-making [18], and visual word recognition [19].

Along with different OrthoR, the spatial pattern between scripts affects cortical activation on neurolinguistic fMRI and Korean script system provides an excellent opportunity to identify the effect of VSF given that the pair of Korean Hangul and Hanja has a unique property of very similar spatial pattern. Meta-analysis demonstrated different visual word form area (VWFA) of peak response and amplitude between LogoS of Chinese and PhonoS of the alphabetic script [20]. However, the Hangul was packaged into “square” units of several phonemes, representing a syllable, with the same visual appearance as Hanja and showed no significant difference of within-subject design of early Chinese-Korean bilinguals between LogoS of Chinese and PhonoS of Korean [21]. Also, because of the government policy, the younger Korean generation was exposed to the Hanja beginning in middle school, which leads to control of the age of language acquisition, consequently, to avoid potential confounders inducing different neural activation [12, 22].

Hence, the purpose of this study was to identify the extent and degree of which VSF affects cortical representation for Hangul versus Hanja as PhonoS versus LogoS, respectively, given different OrthoR. The native Koreans with a first language (L1) of PhonoS and a second language (L2) of LogoS were divided into high-competent (High-C) versus low-competent (Low-C) groups for L2 of LogoS. We hypothesized that the relative higher activation in Low-C group, if any, could more likely be due to neural efficiency such as attention and cognition rather than core neurolinguistic neural demand. The whole brain cortical topology of VSF effect could provide a platform on language processing such as in bilingualism using assimilation versus accommodation between L1 and L2 languages.

## 2. Materials and Methods

### 2.1. Participants

A total of 24 right-handed, native Korean undergraduate students at a local university participated in the fMRI study. The participants were divided into two groups, those familiar with Hanja (High-C group, mean age = 30 ± 5.1 years, *n* = 12, male/female = 6/6) and those not familiar with Hanja (Low-C group, mean age = 29 ± 6.7 years, *n* = 12, male/female = 6/6). All participants learned Hangul as the native language and were familiar with the script. Written consent was obtained from all subjects and the protocol was approved by the Institutional Review Board of Gachon Medical Center. The inclusion criteria for High-C group were subjects having a Hanja certificate above level 2 (high level) awarded by the Korean Association for the Promotion of Hanja Education (KAPHE) and reported daily experience with writing or reading Hanja at least for two recent years before the experiment.

### 2.2. fMRI Paradigm

**Figure 1:**
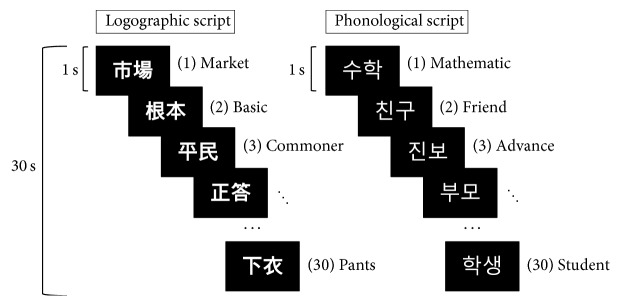
LogoS and PhonoS used for the word reading experiment. LogoS words were selected based on level 5 proficiency guided by the KAPHE, which is equivalent to a middle school educational level in Korea. PhonoS words were chosen based on those most frequently used in daily life. Within each activation block, a word was displayed for 1 s. A total of 30 words were presented in each block.

The fMRI experiment had a block design consisting of Hanja and Hangul trials that were randomly intermixed across the run [5, 6]. A total of 150 two-letter Hanja words were chosen based on KAPHE guidelines for level 5 (middle to low level). To match the Hanja words, a total of 150 of the most frequently used two-letter Hangul words were selected. A Hanja item (e.g., “市場” = market) or a Hangul item (e.g., “
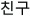
” = friend) was presented for 1 second within the block according to the trial. Each block had 30 items. Subjects were instructed to internally read the Hanja or Hangul word displayed on the screen without actual vocalization (implicit reading) during the fMRI scan. After finishing each block, a fixed cross was displayed for 30 seconds. The run order was randomized for each subject to eliminate systemic bias. One run consisted of 5 blocks and each participant performed two runs. The total duration of the experiment was approximately 30 minutes (Figure 1). After finishing the fMRI experiments, all participants were asked to disclose any Hanja scripts that they could not read during the fMRI task, and no one responded. The words were presented using a beam projector with an 8-inch screen and DMDX software (http://www.u.arizona.edu/~kforster/dmdx/dmdx.htm) [18].

### 2.3. MRI Procedure and Image Analysis

A 3 T whole-body Siemens scanner (Siemens, Magnetom Verio, Erlangen, Germany) was used for image acquisition with an interleaved T2^*∗*^-weighted EPI gradient-echo sequence (TR/TE/*α* = 3000 ms/28 ms/90°, slice thickness = 3 mm, in-plane resolution = 3 × 3 mm^2^, 39 slices, FOV = 192 mm, and matrix size = 64 × 64). Each subject's anatomical image was acquired using a high-resolution (1 × 1 × 1 mm^3^), T1-weighted, 3D gradient-echo pulse sequence (MPRAGE; TR/TE/TI = 1770 ms/2.93 ms/900 ms). fMRI images were analyzed using statistical parametric mapping (SPM 8) (Wellcome Department of Cognitive Neurology, London, UK) running under MATLAB 2008a (Mathworks, Sherborn, MA, USA).

The first five images were discarded from analysis to eliminate the nonequilibrium effects of magnetization. Functional volumes were realigned, coregistered to anatomical images, and normalized to the Montreal Neurological Institute (MNI) template using a transformation matrix acquired from the T1 anatomical image normalization based on the SPM T1 template. Finally, spatial smoothing was performed with an 8 mm full-width at the half-maximum Gaussian kernel. The resulting time-series was high-pass filtered with a cut-off time window of 128 s to remove low-frequency drift in the blood-oxygen level dependent (BOLD) signal. Then, the design matrix was temporally convolved with a canonical hemodynamic response function for a better fit.

A voxel-based general linear model was used at the single subject level to estimate the parameters associated with the conditions of interest (Hanja reading or Hangul reading versus fixed cross as a baseline) along with six motion parameters as covariates of no interest. With the parameters acquired from the first level analysis, a group level analysis was performed using a 2 × 2 factorial design. Group results were estimated by comparing the parameter estimates acquired at the first level analysis. The two contrasts of interest were the main effect analysis used to identify cortical regions where the BOLD response increased during Hanja versus Hangul reading, due to the effect of the script, and the interaction effect analysis used to identify substantially different neural involvement modulated by Hanja script competency. The interaction analysis was confined to the regions with a positive response for Hanja reading compared to Hangul reading at uncorrected *p* = 0.05 (*t*-contrast) to exclude deactivated voxels. Because our primary interest was the regions representing positive responses for Hanja reading compared to Hangul reading and based on combined intensity and cluster size thresholds for the complex cognitive and affective processes which have small effect size, the significance threshold for the main effect and the interaction effect was set at uncorrected *p* < 0.001 and uncorrected *p* < 0.005, respectively (assuming independence, joint probability of type I is 0.005*∗*0.05 = 0.0025, approximately 0.001) with a spatial extent larger than 50 voxels [23, 24]. xjView toolbox (http://www.alivelearn.net/xjview) and automated anatomical labeling (http://www.gin.cnrs.fr/AAL) were used to display the statistical results and report anatomical locations of peak *t*-values.

## 3. Results and Discussion

### 3.1. The Main Effect of Cortical Representation of Orthographic Regularity

**Figure 2:**
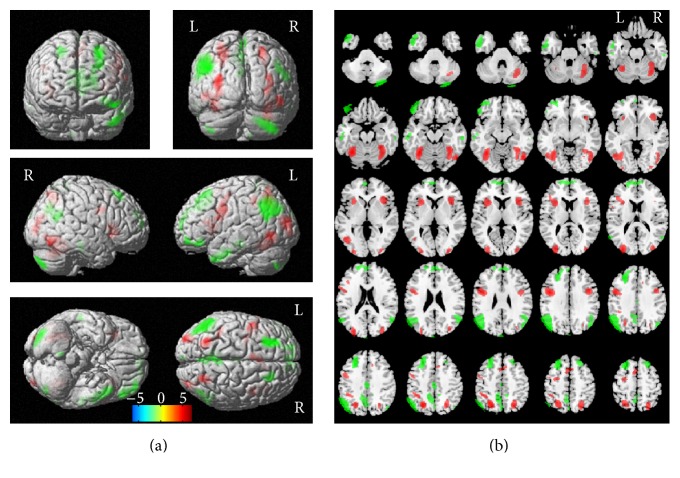
Main effect analysis of script (LogoS versus PhonoS reading) for all subjects (*n* = 24). Red color represents greater BOLD signal for LogoS than for PhonoS reading. Green color represents greater BOLD signal for PhonoS than for LogoS reading. In the 3D rendered image, significantly greater activation for LogoS than for PhonoS reading was observed bilaterally in the inferior occipital/temporal and corresponding dorsal stream extending to the SPL. In comparison, significantly greater activation for PhonoS than for LogoS reading was observed in the bilateral anterior to mid-temporal cortices and the DMPFC (left predominant) (a). T-maps overlaid on sliced images clearly showed that both the FG and insular cortex were more activated for LogoS than for PhonoS reading and the precuneus was more activated for PhonoS than for LogoS reading (b) (uncorrected *p* < 0.001, *K* > 50).

The main effect analysis of script showed increased cortical activation in both High-C and Low-C (*n* = 24) groups at the threshold of uncorrected *p* < 0.001 with a cluster size of 50 voxels. The cortical areas with significantly greater activation for reading Hanja than Hangul reading included the bilateral FG, right inferior temporal gyrus, and corresponding dorsal stream extending to the bilateral superior parietal lobule (SPL), bilateral inferior precentral gyrus (IPcG, left > right), bilateral insula, and supplementary motor area (SMA). In addition to cortical regions, two clusters yielded activation in bilateral cerebellar region 6 (right > left). Significantly greater activation for Hangul than for Hanja reading was observed in the left anterior temporal, AG (left ≫ right)/SMG (left ≫ right), bilateral dorsomedial prefrontal cortex (DMPFC, left > right), left VLPFC, and precuneus (Table 1 and Figure 2).

Different script systems recruit a different cortical involvement during reading written word depending on their OrthoR [25], supported by functional neuroimaging, lesion-based [26, 27], and electrical stimulation studies [28, 29]. The current study confirmed that LogoS was associated with a greater increase in BOLD response in the ventral pathway including the bilateral FG extending to the SPL whereas the dorsal pathway including the AG, SMG, and VLPFC was more activated for PhonoS which is in line with the previous studies except for greater activation in the right SPL extending to the right mid-occipital cortex and right inferior prefrontal cortex as well as left SMA, a similar pattern shown in an illustrative study with the Korean scripts [6].

### 3.2. The Interaction Effect of Cortical Representation of Visual Script Familiarity

**Figure 3:**
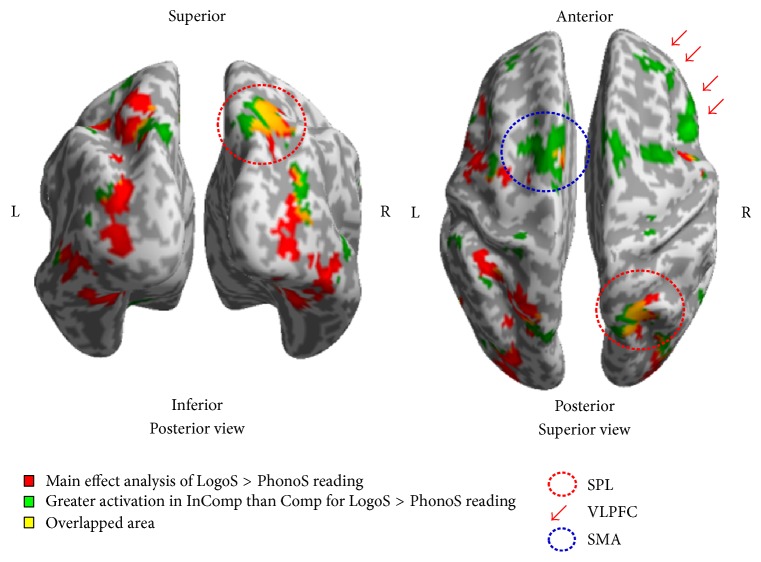
3D surface rendered images of main effect analysis overlaid by the LogoS VSF difference between Low-C and High-C. The 3D rendered images showed the results of the main effect analysis of script overlaid by the greater activation in the Low-C than High-C for LogoS than for PhonoS reading. The overlaid surface rendered images clearly showed the right occipital cortex extending to the right SPL (red dotted circle, posterior and superior view) and right VLPFC (red arrow, superior view) including the SMA (blue dotted circle, superior view) were predominant in the Low-C (uncorrected *p* < 0.001, *K* > 50).

The analysis of the interaction effect showed substantially different cortical activation depending on Hanja competency at the threshold of approximately uncorrected *p* < 0.001 with a cluster size of 50 voxels. Significant greater activation for Hanja than for Hangul reading in the Low-C compared with High-C group occurred in the right SPL, SMA, right VLPFC, and dorsolateral prefrontal cortex. In comparison, areas showing significant greater activation for Hanja than Hangul reading in the High-C compared with Low-C group included the bilateral caudate (right ≫ left) (Table 2 and Figure 3).

Regarding OrthoR, the pairs of Korean Hangul (PhonoS) and Hanja (LogoS) are similar to the pairs of Japanese Kana and Kanji while the Korean writing system has an interestingly unique property. The spatial pattern between Hangul and Hanja script is very similar. Unlike the letters of the Latin alphabet, the Hangul is packaged into “square” units of subsyllable, each of which transcribes a syllable. It is actually composed of several consonant and vowel letters with the form of left to right or from top to down direction. The spatial features of Hanja have geometrical relationship between radicals, which is similar to that of Hangul [4, 6, 21]. The spatial pattern between scripts is essential in brain mapping. fMRI study showed that the anatomical location of neural involvement could be influenced by not only OrthoR but also the spatial pattern between scripts. The authors showed Chinese and English script to Chinese-English bilinguals who were native Chinese speakers fluent in English and English learners of Chinese and showed a location difference of peak response in visual word form area (VWFA) between Chinese and an alphabetic characters such as English [20]. However, evaluating early Chinese-Korean bilinguals using a within-subject design, they reported no significant difference in the location of peak response and amplitude in VWFA between Chinese and Korean script [21].

Additionally, controlling the age at which language acquisition induces different neural activation is needed. Regarding the age of acquisition, a spatially segregated region was found activated in Broca's area but not Wernicke's area in late L2 learners during L1 and L2 reading, whereas this phenomenon was not observed in early L2 learners; they shared a substantially overlapped region (both Broca's area and Wernicke's area) during L1 and L2 reading [12]. Another study also reported the influence of age in L2 exposure and showed increased variability of local brain activity in late L2 exposure [22].

Since the 14th century, Hangul (PhonoS) and Hanja (LogoS) have been the two basic scripts used in Korea, often combined for the purpose of better communication while Hangul and Hanja represent shallow and deep OrthoR, respectively. Also, because of the government policy, younger Korean generation currently is being exposed to Hanja starting in middle school, which leads to control of the age of language acquisition, consequently avoiding the potential confounder inducing different neural activation [12, 22].

To delineate the effect of VSF on the cortical representation, an implicit word reading task was performed for Hangul and Hanja in the subjects with L1 as Hangul divided into High-C versus Low-C group during fMRI acquisition. Compared with High-C group, Low-C group showed greater involvement of the right hemisphere including the right SPL, left SMA, and right IPcG during Hanja compared to Hangul reading. Given Low-C group less proficient in Hanja, the areas of greater activation in the Low-C might not be due to intrinsic linguistic processing such as OrthoR but increased demand for attention and cognition such as VSF [30]. VSF is one of the most influential factors affecting speed, the accuracy of visual word recognition [31, 32], and cortical representation [15, 16, 19, 33] as well as performance on verbal tasks such as picture naming [17] and lexical decision-making [18].

While phonological awareness is closely related to reading and learning PhonoS such as English, due to visual-spatial complexity, visuospatial processing is crucial for reading in LogoS (e.g., Chinese, Korean Hanja) [34]. Several previous studies have suggested that visual skills are more predictive of acquisition and reading in LogoS than PhonoS such as English. A cross-cultural study that examined phonological awareness, visual skills, and reading ability in British, Taiwanese, and Hong Kong children showed that visual skills were significantly related to the reading ability in both Taiwanese and Hong Kong children but not British children, suggestive of clear advantage on the visuospatial skill in reading LogoS [35–37].

The experimental studies suggested that the right parietal cortex has an important role in visuospatial processing such as visuospatial attention and mental rotation of an object in space [38]. In a parametric design with PET, gradually rotated character stimuli from 10% to 70% degree were shown to the participants and the participant were asked to decide whether each stimulus was normal or mirrored. It was demonstrated that the degree of cortical activation in right SPL is incrementally associated with increase of rotational degree of the stimuli [39]. Consistent with other findings, a study comparing patients with right parietal lobe lesions with left and matched normal controls demonstrated that the patient with right parietal lesion revealed low accuracy and delayed response time in constructing mental image rotation task, suggesting the specialization of right SPL in visuospatial processing [40, 41]. Taken together, visual property of the LogoS might cause the Low-C group to require an increase in cognitive demand for spatial processing during LogoS recognition [42, 43]. The SMA is mainly involved in the speech-motor preparation and execution components of reading in a less proficient language. So greater activation in the SMA in the Low-C group might have been due to increased cognitive demand during speech-motor preparation in subjects less proficient in LogoS [44]. In summary, the Low-C group might have required greater cognitive resources for the processing of visuospatial analysis and phonological retrieval by those less practiced and less proficient scripts than the High-C group, which consequently resulted in greater right hemisphere lateralization in the Low-C group. Delineation of neural circuitry concerning VSF on different scripts could be extended to the cortical mapping of acquisition and processing between L1 and L2 languages in bilingualism.

## 4. Conclusions

For cortical mapping to represent Hangul (PhonoS) versus Hanja (LogoS) in the subjects with the first language (L1) PhonoS divided into two groups as high versus low competency to L2 LogoS (High-C versus Low-C), Low-C group demonstrated activation and lateralization in the right SPL and VLPFC as well as left SMA, likely due to neural efficiency such as visuospatial attention and cognition rather than core neurolinguistic neural demand like OrthoR processing.

## Figures and Tables

**Table 1:** BOLD activation for LogoS versus PhonoS reading (*n* = 24).

Contrast	Region	BA	Cluster size	*x*	*y*	*z*	*Z* score
LogoS > PhonoS	Fusiform_L	37	518	−32	−58	−18	5.68
	Occipital_Mid_L	19	589	−32	−88	16	5.17
	Parietal_Sup_L	7	934	−28	−60	48	5.35
	Precentral_L	9	789	−44	2	32	5.37
	Insula_L	13	618	−32	18	12	4.62
	Precentral_L	6	113	−28	−6	54	3.80
	Supp_Motor_Area_L	6	334	−8	4	62	4.15
	Fusiform_R	37	514	34	−52	−16	5.13
	Temporal_Inf_R	37	227	48	−66	−12	5.13
	Parietal_Sup_R	7	890	26	−66	50	4.62
	Insula_R	13	576	34	22	6	4.55
	Precentral_R	9	255	44	4	32	4.95
	Cllm_6_L		160	−32	−54	−24	5.68
	Cllm _6_R		682	32	−62	−26	5.13

PhonoS > LogoS	Occipital_Mid_L	39	1746	−44	−76	32	4.74
	Temporal_Mid_L	21, 38	797	−50	4	−34	4.34
	Frontal_Sup_L	8, 9, 10	2048	−28	34	36	5.16
	Frontal_Inf_Orb_L	11	536	−50	36	−16	4.74
	Cingulum_Mid_L	24	167	0	−18	42	4.11
	Temporal_Mid_R	21	554	56	−64	20	4.34
	Temporal_Inf_R	20, 21	99	62	−26	−18	3.60
	Paracentral_Lobule_R	5	1140	2	−44	66	4.21
	Frontal_Sup_R	8	282	24	30	52	3.89
	Precuneus_R	31	53	12	−42	44	3.59
	Cllm _Crus2_L		113	−44	−78	−48	4.23
	Cllm _Crus2_R		563	30	−92	−38	5.21
	Cllm _9_R		59	8	−58	−64	3.70

**Table 2:** Interaction effect analysis of PhonoS versus LogoS and LogoS competency.

Contrast	Region	BA	Cluster size	*x*	*y*	*z*	*Z* score
LogoS-PhonoS in High-C > LogoS-PhonoS in Low-C	Caudate_R		82	12	16	10	2.99
	Caudate_L			-6	10	8	2.40

LogoS-PhonoS in Low-C > LogoS-PhonoS High-C	Parietal_Sup_R	7	302	20	−64	48	2.62
	Supp_Motor_Area_L	8	788	14	22	40	2.52
	Frontal_Inf_Tri_R	46	64	52	24	28	2.03
	Frontal_Sup_L	6	57	−22	−10	48	2.29
	Frontal_Inf_Tri_L	47	60	−44	24	4	2.58
